# Validation of a French adaptation of the Harvard Trauma Questionnaire among torture survivors from sub-Saharan African countries

**DOI:** 10.3402/ejpt.v3i0.19225

**Published:** 2012-12-06

**Authors:** Capucine de Fouchier, Alain Blanchet, William Hopkins, Eric Bui, Malik Ait-Aoudia, Louis Jehel

**Affiliations:** 1 Centre du Psychotrauma de l'Institut de Victimologie, Paris, France; 2 Laboratoire de Psychopathologie et Neuropsychologie (EA 2027), Université Paris 8, Saint-Denis, France; 3 Freedom from Torture, London, United Kingdom; 4 Laboratoire du Stress Traumatique (EA 4560), CHU de Toulouse & Universite de Toulouse, Toulouse, France; 5 Massachusetts General Hospital & Harvard Medical School; 6 Centre Hospitalier Universitaire de Martinique, Université Antilles Guyane, Fort de France, France; 7 INSERM U669, Laboratoire d’éthique Médicale et de Médecine Légale (EA 4569), Paris, France

**Keywords:** Refugees, ethnic/cultural minorities, torture, cross-cultural assessment, questionnaire

## Abstract

**Background:**

To date no validated instrument in the French language exists to screen for posttraumatic stress disorder (PTSD) in survivors of torture and organized violence.

**Objective:**

The aim of this study is to adapt and validate the Harvard Trauma Questionnaire (HTQ) to this population.

**Method:**

The adapted version was administered to 52 French-speaking torture survivors, originally from sub-Saharan African countries, receiving psychological treatment in specialized treatment centers. A structured clinical interview for DSM was also conducted in order to assess if they met criteria for PTSD.

**Results:**

Cronbach's alpha coefficient for the HTQ Part 4 was adequate (0.95). Criterion validity was evaluated using receiver operating characteristic curve analysis that generated good classification accuracy for PTSD (0.83). At the original cut-off score of 2.5, the HTQ demonstrated high sensitivity and specificity (0.87 and 0.73, respectively).

**Conclusion:**

Results support the reliability and validity of the French version of the HTQ.

In 2010, the United Nations High Commissioner for Refugees identified the United States of America and France as the industrialized countries receiving the highest number of asylum applications per year, 55,500 and 47,800 respectively. Among all the asylum claims lodged in these 44 industrialized countries, 25.5% came from Africa and about 15,000 of them were from countries in which French is the official language (United Nations High Commissioner for Refugees, 2011).

As defined in the United Nations Convention against Torture and Other Cruel, Inhuman or Degrading Treatment or Punishment, torture refers to:

Any act by which severe pain or suffering, whether physical or mental, [which] is intentionally inflicted on a person for such purposes as obtaining from him or a third person information or a confession, punishing him for an act he or a third person has committed or is suspected of having committed, or intimidating or coercing him or a third person, or for any reason based on discrimination of any kind, when such pain or suffering is inflicted by or at the instigation of or with the consent or acquiescence of a public official or other person acting in an official capacity. (United Nations Convention Against Torture, 1987)

The terrifying traumatic experience of torture is a strong risk factor for mental health problems. A variety of psychiatric diagnoses according to the 4th edition of the *Diagnostic and Statistical Manual of Mental Disorders* (DSM) criteria (4th ed., text rev.; *DSM-IV*-TR; American Psychiatric Association, 2000) such as major depressive episode (MDE), posttraumatic stress disorder (PTSD), and generalized anxiety disorder (GAD) have been shown to be associated with torture (Başoğlu, Paker, Özmen, Taşdemir, & Sahin, 1994; Eytan, Durieux-Paillard, Whitaker-Clinch, Loutan, & Bovier, 2007; Holtz, 1998; Maercker & Schützwohl, 1997; Mollica, McInnes, Pham et al., 1998; Mollica, McInnes, Poole, & Tor, 1998; Shrestha et al., 1998). A recent meta-analysis, reviewing 161 articles (pooled sample of *N*=81,866 refugees), reported that the prevalence rates for MDE and PTSD in this population were of 30.8 and 30.6%, respectively (Steel et al., 2009). The authors have also brought to light that when only studies related to torture victims are taken into account, the prevalence of posttraumatic and depressive disorders increases significantly.

French-speaking torture survivors mainly originate from sub-Saharan African countries such as the Democratic Republic of the Congo (DRC), Cameroon, or Ivory Coast. Although about 15,000 sub-Saharan French-speaking African refugees flee their country each year (United Nations High Commissioner for Refugees, 2011), little research has investigated the specific cultural expression of mental health disorders and screening instruments in this population. To the best of our knowledge, only four studies have attempted to describe the traumatic responses and the validity of *DSM-IV*-TR criteria in French-speaking sub-Saharan African populations.

Tang and Fox (2000, 2001) assessed PTSD among 80 Senegalese refugees resettled in the Gambia. In order to do that, participants were assessed with the Harvard Trauma Questionnaire (HTQ) (Mollica et al., 1992). Interpreters were used when the researcher and the participant did not share the same language. The most frequent traumatic events experienced by the participants were “lack of food or water,” “combat situations,” and “being close to death”; “torture” concerned only 13 persons of the sample. Ten percent of the sample scored above the critical cutoff of 2.5 for PTSD. The most frequent items endorsed were those reflecting reexperiencing and increased arousal, while those assessing ‘‘the inability to remember parts of the most traumatic or hurtful event(s)’’ and ‘‘numbing’’ were only endorsed by 2% of the participants.

Fox (2003) compared clinical descriptions of traumatic responses given by traditional healers to the concept of PTSD and showed that some of the symptoms described are consistent with PTSD; specifically hyperarousal, flashbacks, nightmares, difficulty concentrating, and avoidance behaviors. However, other symptoms described such as “body heat”, “sitting,” and “staring” did not fit the diagnosis.

A third study examining the validity of PTSD among “Bushmen” belonging to hunting community in the Kalahari desert (McCall & Resick, 2003) suggested that PTSD diagnosis as defined by *DSM-IV*-TR was appropriate for this specific African population. All participants (*N*=20 victims of violent assault) endorsed ‘‘reliving the trauma’’, ‘‘markedly diminished interest or participation in significant activities,’’ and ‘‘restricted range of affect’’, while symptoms of avoidance and increased arousal, and numbing were only rarely endorsed.

In a more recent study, Rasmussen, Smith, and Keller (2007) explored the structure of PTSD symptoms in Central and Western African population. Asylum seekers from those regions (*N*=400), living in the Unites States, were asked to complete the HTQ with the help of interpreters. The authors found high levels of intrusion (53 to 81%) and avoidance symptoms (64 to 67%), followed by arousal symptoms (47 to 67%), and low rates of numbing symptoms (19 to 39%). Confirmatory factor analysis highlighted a comprehensive model of PTSD in this population including four factors: aroused intrusion, numbing, avoidance, and hypervigilance. Of interest, the strongest association was found between aroused intrusion and symptoms of hypervigilance while the weakest was found between avoidance and numbing.

Taken together, these prior studies suggest that PTSD symptoms as defined by the *DSM-IV*-TR may be used in African individuals; however, they may not account for all the posttraumatic psychopathology/responses in this population. Although evidence suggests that in addition to *DSM-IV*-TR symptoms, culturally specific symptoms might also result from trauma exposure in sub-Saharan African victims of tortures (Fox, 2003; Fox & Tang, 2000; Tang & Fox, 2001), to date, no validated instrument is available to use as a screening tool in this mainly French-speaking population.

The American care center “Harvard Program in Refugee Trauma” (HPRT) was a pioneer in the development and adaptation of tools for psychiatric evaluation, which take into consideration the degree and type of trauma experienced by survivors of torture and organized violence. To this end, they developed the HTQ.

The HTQ is an instrument built to evaluate torture and the scale of psychological impact (Mollica et al., 1992) in inter-cultural contexts. Originally created for the Indochinese populations living in America, this questionnaire was then adapted for other non-western refugee populations such as Russian, Urdu, Arabic, Sri Lankan, Bosnian, and Tibetan (Halepota & Wasif, 2001; Kleijn, Hovens, & Rodenburg, 2001; Lhewa, Banu, Rosenfeld, & Keller, 2007; Miyazaki, Dewaraja, & Kawamura, 2006; Mollica et al., 1992; Oruc et al., 2008; Shoeb, Weinstein, & Mollica, 2007). However, a validated French version does not exist in the current literature. The aim of this research is therefore to adapt and validate a French version of the HTQ for French-speaking refugees and asylum seekers coming from sub-Saharan African countries.

## Method

### Participants

The sample consisted of 52 French-speaking torture survivors, originally from sub-Saharan African countries, who received care in four specialized treatment centers: The Medical Foundation for the Care of the Victims of Torture in England (*n*=22), the Psychotrauma Centre of the Victimology Institute (*n*=17) in France, the France Terre D'Asile induction centre (*n*=7), and Tenon University Hospital (*n*=6) in France (Table 1). They gave oral informed consent to their therapist or social worker, if they agreed to participate; this information was then recorded in each participant's medical file by the principal investigator.

**Table 1:** Socio-demographic characteristics of the study sample (*N*=52)

	Female		Male		Total	
			
	*N*	%	*N*	%	*N*	%
Marital status						
Single	14	60.9	17	58.6	31	59.6
Married	8	34.8	12	41.4	20	38.5
Widowed once	1	4.3	0	0.0	1	1.9
Education						
Primary	6	26.1	1	3.4	7	13.5
Secondary	5	21.7	7	24.1	12	23.1
High school	8	34.8	8	27.6	16	30.8
University	4	17.4	13	44.8	17	32.7
Asylum situation						
Pending	5	21.7	12	41.4	17	32.7
Refugee status	8	34.8	6	20.7	14	26.9
Appeal	7	30.4	8	27.6	15	28.8
Refusal	3	13.0	3	10.3	6	11.5
Psychotropic medication						
Yes	16	69.6	19	65.5	35	67.3
No	7	30.4	10	34.5	17	32.7

Of the 52 participants, 56% were men with an average age of 36.6 (SD=6.47), respectively, from the DRC (*n*=18), Ivory Coast (*n*=12), Cameroon (*n*=12), Guinea (*n*=5), Rwanda (*n*=3), and Mali (*n*=2).

Sixty percent of the participants were single, with a high level of education since 63.5% of them have completed high school.

The participants all lodged an asylum application when they arrived in England or France, countries in which they had spent an average of 35.7 months (SD=26.59) at the time of the research. In terms of their legal immigration status, 26.9% had obtained a refugee status or humanitarian leave to remain, 32.7% were still waiting for a decision, 28.8% were appealing the initial refusal, and 11.5% had been refused by court.

The traumatic events that were the most frequently reported by the participants were beating to the body (90%), torture (77%), rape (52%), other types of sexual abuse or sexual humiliation (56%), forced to hide (63%), or other separation from family members (58%).

At the time of the research, 67.3% of the participants were receiving psychotropic medication, 50% were taking anti-depressants, 51.9% were under anti-anxiety drugs, and 3.8% were taking anti-psychotic medications.

### Adaptation and translation

The adaptation and translation methodology was designed following the HPRT's recommendations (Mollica, McDonald, Massagli, & Silove, 2004) and inspired by Flaherty's methodology (1988) in developing instruments for cross-cultural research.

#### Adaptation

A group of experts was established to assess the content validity of the instrument. The group of experts involved two English-French-Lingala interpreters originally from the DRC, an English psychiatrist, and a French clinical psychologist working at the Medical Foundation for the Care of the Victims of Torture. The expert group validated the entire version of the HTQ as being relevant for the sub-Saharan African culture and added one extra item to the torture descriptions: “being forced to empty toilets and clean them with bare hands,” an act of humiliation that is frequently reported by survivors from this area.

#### Translation

The Brislin's back-translation method (1970) was used for the translation of the instrument. First, a bilingual clinical psychologist translated the version that had been adapted from the English (A) into French. This translation was then evaluated separately by two interpreters, members of the original panel. After which a general consensus was found for each item of the French (B) version.

Third, the “consensus” version (B) was back-translated by two other bilingual (French/English) persons, who had no previous knowledge of the original version (A). Fourth, the concordance between the translation and the back-translated version was evaluated according to a precise scale (3=*same meaning in the two versions*, 2=*almost the same meaning in the two versions*, and 1=*different meaning in the two versions*) by two other bilingual individuals: a French/English clinical psychologist and a French/English interpreter.

Finally, the expert panel as well as the back-translation interpreters met to incorporate the comments of the assessors.

### Measures

#### Harvard Trauma Questionnaire

The HTQ is a five-section self-report questionnaire. The first part lists a series of traumatic events (41) to which the individual subject will answer *yes* or *no*. In the second section, comprising of two open-ended questions, the subject is asked to describe in more detail the event that he found the most traumatizing, whether in his country of origin or since in exile. The third part assesses the risk of neurological complications that may result from certain traumatic events. The fourth part consists of 40 items assessing the psychological impact. The participant is asked to rate each item on a four-point Likert scale (1=*not at all*, 2=*a little*, 3=*quite a bit*, and 4=*extremely*). The first 16 items such as “recurrent thoughts or memories of the most hurtful or terrifying events” or “feeling as though the event is happening again” attempt to assess the accepted symptoms of PTSD diagnosis (Part 4 – PTSD). The following 24 items, named by the authors “refugee-specific”, lean more toward the impact that the traumatic experiences may have had on the subject's perception of his/her daily lives (Part 4 – Functioning). The fifth and final section offers a list of 29 acts that are considered to be torture.

The validation study of the original version was conducted among 91 patients originally from Cambodia, Laos, and Vietnam, receiving treatment at the Indochinese Psychiatric Clinic. Reliability and validity were determined by comparing the results of the questionnaire to the clinician's DSM III-R diagnosis elaborated with a semi-structured interview. The results of this study show that the internal consistency for Part I (0.90) and for Part IV (0.96) was robust. Total item-score correlation coefficients were 0.56 for Part I and 0.65 for Part IV. Furthermore, the criterion validity study showed, for a threshold of 2.5, a sensitivity of 0.78 and a specificity of 0.65 (Mollica et al., 1992; Mollica et al., 1996).

#### Structured clinical interview for DSM

The structured clinical interview for DSM (SCID) (First, Spitzer, Gibbon, & Williams, 2002), a semi-structured diagnostic interview, is considered in international literature as a gold-standard in intercultural validation studies (Mollica et al., 2004). Also, it was used in several validation studies of instruments in refugee populations (Caspi, Carlson, & Klein, 2007). The psychometric properties of the SCID were assessed in numerous studies (Zanarini & Frankenburg, 2001), which showed good inter-raters reliability (from 0.54 and 0.84) and test–retest reliability (between 0.43 and 0.67 in the studies). Because no French version of the SCID has been validated so far in the literature, we used a French version that was translated by the psychiatric department of the Institut Mutualiste Montsouris of Paris, France. Despite the lack of validation of the French version of the SCID, this diagnostic tool was selected because it is considered the gold standard in intercultural context studies (Mollica et al., 2004) and also because it was used in prior studies (Pez et al., 2010; Stip, Caron, Renaud, Pampoulova, & Lecomte, 2003; Weber Rouget et al., 2005).

#### Global Assessment of Functioning

The Global Assessment of Functioning (GAF) is the *DSM-IV*-TR's fifth axis that assesses an individual's psychological, social, and occupational functioning (4th ed., text rev.; *DSM-IV*-TR; American Psychiatric Association, 2000). It locates subjectively the subject along a continuum starting from 1 to 100, with 1 representing the most extreme pathology and 100 representing a person who is symptoms free and has satisfactory social functioning. The scale is further divided into 10 intervals. Studies have shown that the GAF has good psychometric qualities (Hilsenroth et al., 2000; Jones, Thornicroft, Coffey, & Dunn, 1995; Ramirez, Ekselius, & Ramklint, 2008) as well as its validity as a good transcultural instrument (Gaite et al., 2005).

### Procedure

The validation methodology was designed following the HPRT's recommendations (Mollica et al., 2004). The experiment took place in the four different treatment centers from March 2008 until August 2009. Participants were included in the study if they met the following criteria: male or female aged 18 and above, torture survivors, having French as a first or second language, and the ability to provide informed consent. Participants who presented psychotic or cognitive disorders were excluded from the panel. All the enrolled participants met the experimenter in a face-to-face interview during which time a consent form was signed, the PTSD module of SCID was delivered, the GAF was assessed, and the French version of the HTQ was completed. The assessment started first with the SCID in order to minimize memorization bias, which may have influenced the participant's answers.

#### Missing values

A research protocol relating to missing data of the qualitative parts of the HTQ has been conducted in the present study. The analysis of missing data gave an insight into the specificity and accuracy of the post-traumatic events experienced by this specific population.

#### Data analyses

Concurrent validity coefficients were obtained using Pearson's correlations, and internal consistency was calculated using Cronbach's alpha. Criterion validity with PTSD diagnosis according to the SCID was assessed using a receiver operating characteristic (ROC) curve. All analyses were carried out using SPSS 17.0, with an alpha level of significance set at 0.05 (two-tailed).

## Results

Our study consisted firstly in the evaluation of the relevance of the qualitative items of the scale (Part 1, 2, 3, and 5), and, secondly, in examining the psychometric properties of the quantitative parts (Part 4) of the French version. The descriptive statistics for the sample (*M*, SD, α) of the French adaptation of the HTQ are shown in Table 2.

**Table 2:** Descriptive statistics for the French version of the HTQ socio-demographic characteristics of the study sample (*N*=52)

	*M*	SD	α
Part 1 – Traumatic events	14.04	6.53	0.86
Part 4 – PTSD	2.84	0.66	0.90
Part 4 – Functioning	2.67	0.69	0.93
Part 4 – Total	2.74	0.66	0.96
Part 5 – Torture	9.83	5.62	0.88

### Qualitative sections (Part 1, 2, 3, and 5)

The reliability of the qualitative section was assessed using Cronbach's coefficients alpha and were 0.86, 0.79, and 0.88 for the traumatic events section, the head injury section, and the torture section, respectively. But in order to test the relevance of the items in this specific population, items that combined a high level of negative answers and missing values were examined. Thirteen items in the first section, the entire second section, no items of the third section, and two items in the fifth section fulfilled the condition of having a missing values ratio higher than a positive answer ratio.

### Quantitative part (Part 4)

#### Reliability

The coefficient alpha was α=0.90 for the PTSD subscale, α=0.93 for the self-perception of functioning subscale, and α=0.93 for the entire scale (α=0.96).

Item-total scale correlations displayed two items with a correlation below 0.40 (see Table 3, items in bold): “inability to remember parts of the most hurtful or traumatic events” (*r*=0.31; *p*=0.023) and “avoiding thoughts” or “feelings associated with the traumatic or hurtful events” (*r*=0.27; *p*=0.051).

**Table 3:** Descriptive statistics for the quantitative parts of the HTQ Part 4-Total

	*M*/SD	Corrected item-total correlations
Recurrent thoughts or memories of the most hurtful or terrifying events	3.06/0.94	0.51
Feeling as though the event is happening again	2.81/1.07	0.62
Recurrent nightmares	2.87/0.93	0.67
Feeling detached or withdrawn from people	2.88/1.15	0.75
Unable to feel emotions	2.50/1.21	0.68
Feeling jumpy, easily startled	2.87/1.03	0.65
Difficulty concentrating	2.96/0.97	0.78
Trouble sleeping	3.00/1.06	0.68
Feeling on guard	3.08/1.01	0.65
Feeling irritable or having outbursts of anger	2.73/1.08	0.66
Avoiding activities that remind you of the traumatic or hurtful event	3.10/0.89	0.63
Inability to remember parts of the most hurtful or traumatic events	1.87/1.07	0.31
Less interest in daily activities	2.65/1.01	0.58
Feeling as if you don't have a future	3.02/1.18	0.61
Avoiding thoughts or feelings associated with the traumatic or hurtful events	2.92/0.99	0.32
Sudden emotional or physical reaction when reminded of the most hurtful or traumatic events	3.10/0.91	0.72
Feeling that you have less skills than you had before	3.01/0.98	0.66
Having difficulty dealing with new situations	2.78/0.99	0.66
Feeling exhausted	2.66/1.09	0.81
Bodily pain	2.67/1.18	0.60
Troubled by physical problem(s)	2.72/1.16	0.58
Poor memory	2.67/1.09	0.72
Finding out or being told by other people that you have done something that you cannot remember	2.20/1.1	0.70
Difficulty paying attention	2.49/0.94	0.60
Feeling as if you are split into two people and one of you is watching what the other is doing	2.32/1.19	0.51
Feeling unable to make daily plans	2.67/1.13	0.82
Blaming yourself for things that have happened	2.29/1.31	0.65
Feeling guilty for having survived	2.56/1.25	0.59
Hopelessness	3.04/1.05	0.72
Feeling ashamed of the hurtful or traumatic events that have happened to you	3.24/1.02	0.62
Feeling that people do not understand what happened to you	3.10/1.03	0.58
Feeling others are hostile to you	2.56/1.23	0.58
Feeling that you have no one to rely upon	2.65/1.12	0.48
Feeling that someone you trusted betrayed you	2.15/1.19	0.45
Feeling humiliated by your experience	3.13/1.05	0.49
Feeling no trust in others	2.78/1.14	0.54
Feeling powerless to help others	2.35/1.27	0.66
Spending time thinking why these events happened to you	3.37/0.88	0.59
Feeling that you are the only one that suffered these events	2.56/1.23	0.54
Feeling a need for revenge	2.18/1.30	0.52

#### Validity of the HTQ

The criterion validity was assessed using a ROC curve analysis (see Table 4). The area under the curve, the predictive positive, and negative values are equal to 0.83, 0.92, and 0.61, respectively. At cutoff 2.5, the sensitivity and specificity equal 0.87 and 0.73, respectively (see Table 5).

**Table 4:** Cross-tabulation of SCID PTSD vs. HTQ Part 4 identified cases at standard 2.5 cutoff

	SCID PTSD		
	
HTQ Part 4 – PTSD	No, *N* (%)	Yes, *N* (%)	Total, *N* (%)
<2.5	8 (15)	5 (10)	13 (25)
>2.5	3 (6)	36 (69)	39 (75)
Total	11 (21)	41 (79)	52 (100)

**Table 5:** Receiver Operating Characteristic (ROC) analysis of HTQ Part 4-PTSD vs. SCID-I PTSD module analysis of HTQ Part 4-PTSD vs. SCID-I PTSD module

Cutoff	Sensitivity	Specificity
2.09	0.93	0.45
2.16	0.90	0.45
2.25	0.90	0.64
2.34	0.90	0.73
2.45	0.88	0.73
2.55	0.85	0.73
2.59	0.83	0.73
2.65	0.73	0.81
2.7	0.71	0.81

Comparing the result given by the Part 4-Total to the diagnostic given by the SCID, it was found that sensitivity and specificity scored 0.85 and 0.73, respectively.

Correlation coefficient between the Part 4-Functioning (items 17 to 40), described by the authors as an evaluation of the client's perception of his actual functioning, and the GAF was assessed (*r*=− 0.588; *p* < 0.001).

## Discussion

When compared with the results of the original version of the questionnaire (Mollica et al., 1992; Mollica et al., 1996) and the criteria's given by the authors (Mollica et al., 2004), these results provide evidence for the reliability and validity of the French version of the HTQ but raise questions regarding the relevance of a few items.

In the qualitative parts (Parts 1 and 2), the high rates of missing values as well as the participants’ reporting that the questionnaire was too long and overwhelming emotionally, was a significant concern. This forced us to consider an adaptation of the 13 items in question to optimize acceptability of the measure. As a result, we have simplified the questions by generalizing several question options into one specific question. For example, items 25 (“being forced to physically harm a member of your family or a friend”) and 26 (“being forced to physically harm someone who is neither a member of your family nor a friend”) describe two scenarios with the same outcome. Therefore, we decided to combine these two questions in one question, which expressed the essential idea of “being forced to harm someone physically.”

The items of Part 1 which were not transformed and the complete Part 2, which represented over 70% of the missing values, as well as two items from Part 5 (“administration of drugs” and “needles under finger or toe nails”) were removed as they did not seem to add relevant information regarding victims of torture originating from sub-Saharan Africa. An added benefit of this was that the time to complete the questionnaire was considerably reduced, and the responders found the material better adapted to their particular circumstances.

Regarding the statistical evaluation of Part 4 of the HTQ's PTSD subscale, it indicated good diagnostic accuracy as the area under the curve is prior to 0.70 (Mollica et al., 2004). Predictive positive and negative values are high, which shows the strong probability that the results given by the scale might be correct.

Our results provide support for the good statistical validity at the 2.5 threshold, as they are equal if not superior to those found during the validation of the original version, suggesting that the original cutoff of the HTQ is acceptable to assess PTSD in French-speaking torture survivors.

However, it is interesting to note that two items weaken the evaluation's reliability. Item 12, “inability to remember important parts of the painful and traumatic events,” seems to function differently from other items on the scale. This observation is in line with the results from studies on PTSD in French-speaking sub-Saharan African populations (McCall & Resick, 2003; Rasmussen et al., 2007; Tang & Fox, 2001), which suggest that this item does not appear to be a culturally revealing symptom of trauma in sub-Saharan African populations.

Item 15, “avoiding thoughts or feelings associated with painful or traumatic events,” also proved problematic. This question seems to be testing for cognitive avoidance, but it does not correlate well with the total score obtained for PTSD. Normally a high score would be expected but this was not the case. Perhaps one of the reasons for this is the intensity of the trauma and high level of symptoms severity in the current sample group, which renders the figures for cognitive avoidance often related to PTSD to be somewhat weaker than usual. This may lend support to the strong correlation between the PTSD score and behavioral avoidance, which is being similarly tested in item 11. An explanation for this might be that the level of awareness manifested by the responders differs in terms of behavior and cognition. For example, it might be easier to recognize behavioral triggers and the consequences than cognitive processes that may well be happening on an implicit level.

Although these two items affected the evaluation's reliability, their removal from the scale did not result in an increase of the internal consistency of the PTSD subscale and, therefore, it was decided to retain them.

The “functioning” part of section 4, which correlates well with the evaluation of the DSM's fifth axis, seems to be in line with what the authors’ suggests might be expected (i.e., a measure of the subject's perception of his/her actual life experience).

It is also interesting to note that the comparison between the result given by the Part 4-Total to the diagnostic given by the SCID gives good diagnostic accuracy, which is almost equivalent with the one generated by Part 4-PTSD.

The global results for Part 4 of the HTQ, which seem to successfully identify people suffering from PTSD, also confirm the strong links between the intensity of the traumatic pathology and the impact on the subject's self-perception. The principal aim of this study was to offer valid tools to measure trauma in French-speaking torture victims. Though the limitations of this study are inherent in small sample size and the combination of different populations from sub-Saharan Africa, it seems appropriate to conclude that the French adaptation of the HTQ is both reliable and valid.
